# Chemical Genomics Profiling of Environmental Chemical Modulation of Human Nuclear Receptors

**DOI:** 10.1289/ehp.1002952

**Published:** 2011-05-04

**Authors:** Ruili Huang, Menghang Xia, Ming-Hsuang Cho, Srilatha Sakamuru, Paul Shinn, Keith A. Houck, David J. Dix, Richard S. Judson, Kristine L. Witt, Robert J. Kavlock, Raymond R. Tice, Christopher P. Austin

**Affiliations:** 1 National Institutes of Health Chemical Genomics Center, National Institutes of Health, Department of Health and Human Services, Bethesda, Maryland, USA; 2 National Center for Computational Toxicology, Office of Research and Development, U.S. Environmental Protection Agency, Research Triangle Park, North Carolina, USA; 3 National Toxicology Program, National Institute of Environmental Health Sciences, National Institutes of Health, Department of Health and Human Services, Research Triangle Park, North Carolina, USA

**Keywords:** assay performance, chemical genomics, cytotoxicity, nuclear receptors, qHTS, Tox21

## Abstract

Background: The large and increasing number of chemicals released into the environment demands more efficient and cost-effective approaches for assessing environmental chemical toxicity. The U.S. Tox21 program has responded to this challenge by proposing alternative strategies for toxicity testing, among which the quantitative high-throughput screening (qHTS) paradigm has been adopted as the primary tool for generating data from screening large chemical libraries using a wide spectrum of assays.

Objectives: The goal of this study was to develop methods to evaluate the data generated from these assays to guide future assay selection and prioritization for the Tox21 program.

Methods: We examined the data from the Tox21 pilot-phase collection of approximately 3,000 environmental chemicals profiled in qHTS format against a panel of 10 human nuclear receptors (AR, ERα, FXR, GR, LXRβ, PPARγ, PPARδ, RXRα, TRβ, and VDR) for reproducibility, concordance of biological activity profiles with sequence homology of the receptor ligand binding domains, and structure–activity relationships.

Results: We determined the assays to be appropriate in terms of biological relevance. We found better concordance for replicate compounds for the agonist-mode than for the antagonist-mode assays, likely due to interference of cytotoxicity in the latter assays. This exercise also enabled us to formulate data-driven strategies for discriminating true signals from artifacts, and to prioritize assays based on data quality.

Conclusions: The results demonstrate the feasibility of qHTS to identify the potential for environmentally relevant chemicals to interact with key toxicity pathways related to human disease induction.

Fast and accurate evaluation of environmental chemical toxicity currently is challenged by the high cost and low-throughput nature of traditional toxicity testing methods and the large number of environmental chemicals that need to be evaluated [Dix et al. 2007; Kavlock et al. 2009; National Research Council (NRC) 2007]. In response to these challenges, three government agencies—the U.S. Environmental Protection Agency (EPA), the National Toxicology Program (NTP), and the National Institutes of Health (NIH) Chemical Genomics Center (NCGC)—launched a collaborative initiative, the Tox21 program (Collins et al. 2008; Kavlock et al. 2009), and were recently joined by the U.S. Food and Drug Administration. The goal of this effort is to employ a broad spectrum of *in vitro* assays to use high-throughput screening (HTS) methods to screen a large number of environmental chemicals for their potential to disturb biological pathways that may result in toxicity. The data generated will be used to derive biological and chemical profiles that could serve as the basis for prioritization of chemicals for further toxicological evaluation (Reif et al. 2010), act as predictive surrogates for *in vivo* toxicity end points (Judson et al. 2010), and generate testable hypotheses on mechanism of toxicity (Huang et al. 2008; Xia et al. 2009b).

Nuclear receptors (NRs) are a family of transcription factors that are important regulators of metabolism, differentiation, apoptosis, and cell cycle progression. The transcriptional activities of NRs are regulated by small, lipophilic molecules (Gronemeyer et al. 2004), including pharmaceutical agents and chemicals in the environment, and their altered function has been related to a number of diseases (Kersten et al. 2000; Sonoda et al. 2008; Tontonoz and Mangelsdorf 2003). For example, interaction of a variety of pesticides and other industrial chemicals with the estrogen and androgen NRs has been linked to a number of adverse health consequences, including birth defects, impaired reproductive capacity, developmental neurotoxicity, and certain cancers (Damstra et al. 2002). Because the mechanism of action leading to such toxicities is directly linked to chemicals binding to NRs, they make an ideal starting point for using HTS tools to characterize toxicity pathways as envisioned by the NRC (2007). As a Tox21 proof-of-concept study, we screened an environmentally relevant library consisting of approximately 3,000 chemicals against a panel of 10 human NRs—the androgen receptor (AR), estrogen receptor α (ERα), farnesoid X receptor (FXR), glucocorticoid receptor (GR), liver X receptor β (LXRβ), peroxisome proliferator-activated receptor γ (PPARγ), peroxisome proliferator-activated receptor δ (PPARδ), retinoid X receptor α (RXRα), thyroid hormone receptor β (TRβ), and vitamin D receptor (VDR)—in a quantitative high-throughput screening (qHTS) format (Inglese et al. 2006; Xia et al. 2009a). In this format, a concentration–response curve is generated for every compound to identify both potential agonists and antagonists.

The systematic profiling of a large set of environmental chemicals such as the Tox21 compound collection against the panel of 10 NRs is the initial step toward identifying substances with endocrine-disrupting and other NR-mediated toxicity potential. We examined the interactions for concordance with expected findings for a small number of well-characterized NR ligands, for reproducibility across duplicate chemicals in the library, for biological profiles by clustering activities across NRs based on sequence homology of their ligand-binding domains (LBDs), and by phenotypic clustering to look for structure–activity relationships (SARs). The results demonstrate the feasibility of HTS to identify the potential for environmentally relevant chemicals to interact with key toxicity pathways related to human disease induction.

## Materials and Methods

*Compound collection.* The current Tox21 compound collection consists of 2,870 compounds: 1,408 provided by the NTP (Xia et al. 2008) and 1,462 provided by the U.S. EPA (Huang et al. 2009; Judson et al. 2009). The structures and annotations of these compounds are publicly available (Huang 2010; PubChem 2007, 2009). The compounds were dissolved in dimethyl sulfoxide (DMSO) and plated in 1,536-well plate format at 14 or 15 concentrations ranging from 0.1 μM to 20 mM. See Supplemental Material for more details (doi:10.1289/ehp.1002952).

β*-Lactamase reporter gene assay and qHTS.* GeneBLAzer β-lactamase (*bla*) HEK 293T cell lines that constitutively co-express a fusion protein composed of the LBDs of related human NRs coupled to the DNA-binding domain of the yeast transcription factor GAL and cell culture reagents were obtained from Invitrogen (Carlsbad, CA). See Supplemental Material for more details (doi:10.1289/ehp.1002952).When activated, these fusion proteins then stimulate *bla* reporter gene expression. Compound formatting and qHTS were performed as described previously (Xia et al. 2009b). Briefly, the *bla* cells with different NRs were dispensed in 1,536-well plates for screening. After cells were incubated for 5–6 hr, compounds at 14 or 15 concentrations from the NTP and U.S. EPA collections were transferred to the assay plate with the final concentrations ranging from 0.5 nM to 92 μM. The plates were incubated for 16–18 hr at 37°C before detection mix was added, and the plates were then incubated again at room temperature for 1.5–2 hr. Fluorescence intensity (405 nm excitation, 460- and 530-nm emission) was measured using an Envision plate reader (PerkinElmer, Shelton, CT). Data were expressed as the ratio of 460-nm to 530-nm emissions. The assay performance was assessed by plate statistics (signal-to-background ratio, Z´-factor, coefficient of variation) (Zhang et al. 1999) (see Supplemental Material, Table 2).

**Table 1:** NR ligands and their observed assay activities.

NR ligand name	Known NR activity*a*	NR assay*b*	Observed phenotype*c*	Observed EC_50_ or IC_50_ (μM)*d*
Danazol		AR agonist		AR agonist		Activator		0.06
17α-Hydroxyprogesterone		AR agonist		AR agonist		Activator		0.002
Progesterone		AR agonist		AR agonist		Activator		0.06
Corticosterone		AR agonist		AR agonist		Activator		0.22
Cyproterone acetate		AR antagonist		AR antagonist		Inhibitor		15.85
Flutamide		AR antagonist		AR antagonist		Inhibitor		31.62
Mifepristone		AR antagonist		AR antagonist		Inhibitor		2.00
Nilutamide		AR antagonist		AR antagonist		Inhibitor		12.59
Diethylstilbestrol		ERα agonist		ERα agonist		Activator		< 0.001
β-Estradiol		ERα agonist		ERα agonist		Activator		< 0.001
1,3,5-tris(4-Hydroxyphenyl)-4-propyl-1H-pyrazole		ERα agonist		ERα agonist		Activator		0.10
4-Hydroxytamoxifen		ERα antagonist		ERα antagonist		Inhibitor		0.07
Raloxifene hydrochloride		ERα antagonist		ERα antagonist		Inhibitor		0.03
Tamoxifen citrate		ERα antagonist		ERα antagonist		Inhibitor		8.91
Lithocholic acid		FXR agonist		FXR agonist		Activator		22.39
Beclomethasone		GR agonist		GR agonist		Activator		0.04
Betamethasone		GR agonist		GR agonist		Activator		0.09
Dexamethasone		GR agonist		GR agonist		Activator		0.02
Hydrocortisone		GR agonist		GR agonist		Activator		0.08
Triamcinolone		GR agonist		GR agonist		Activator		0.11
Cholesterol		LXR agonist		LXRβ agonist		Activator		39.81
TO-901317		LXR agonist		LXRβ agonist		Activator		0.25
Fmoc-l-leucine		PPARγ agonist		PPARγ agonist		Activator		11.22
Ciglitizone		PPARγ agonist		PPARγ agonist		Activator		1.41
GW1929		PPARγ agonist		PPARγ agonist		Activator		0.002
Troglitazone		PPARγ agonist		PPARγ agonist		Activator		0.13
DRF 2519		PPARγ agonist		PPARγ agonist		Activator		0.10
BADGE		PPARγ antagonist		PPARγ antagonist		Inhibitor		50.12
GW9662		PPARγ antagonist		PPARγ antagonist		Inhibitor		12.59
2-Bromohexadecanoic acid		PPARδ agonist		PPARδ agonist		Activator		28.18
GW0742		PPARδ agonist		PPARδ agonist		Activator		19.95
l-165,041		PPARδ agonist		PPARδ agonist		Activator		0.09
13-*cis*-Retinoic acid		RAR agonist		RXRα agonist		Activator		0.25
TTNPB		RAR agonist		RXRα agonist		Activator		0.71
AM-580		RARα agonist		RXRα agonist		Activator		0.62
Retinoic acid		RXR agonist		RXRα agonist		Activator		0.13
3,3´,5-Triiodo-l-thyronine		TR agonist		TRβ agonist		Activator		< 0.001
Lithocholic acid		VDR agonist		VDR agonist		Activator		22.39
Abbreviations: BADGE, bisphenol A diglycidyl ether; RAR, retinoic acid receptor; TTNPB, (E)-4-[2-(5,6,7,8-tetrahydro-5,5,8,8-tetramethyl-2-naphthylenyl)-1 -propenyl] benzoic acid. **a**NR activity of compound reported in literature. **b**The NR *bla* assay in which the compound was tested. **c**Activity observed for the compound when tested in the NR *bla* assay. **d**EC_50_ (agonist) or IC_50_ (antagonist) obtained for the compound when tested in the NR *bla* assay.								

*qHTS data analysis.* Analysis of compound concentration–response data was performed as previously described (Inglese et al. 2006). See Supplemental Material for more details (doi:10.1289/ehp.1002952). Briefly, raw plate reads for each titration point were first normalized relative to the positive control compound and DMSO-only wells and then corrected by applying an NCGC in-house pattern correction algorithm using compound-free control plates (i.e., DMSO-only plates) at the beginning and end of the compound plate stack. Concentration–response titration points for each compound were fitted to a four-parameter Hill equation (Hill 1910), yielding concentrations of half-maximal activity (AC_50_) and maximal response (efficacy) values. Compounds were designated as class 1–4 according to the type of concentration–response curve observed (Inglese et al. 2006). Curve classes are heuristic measures of data confidence, classifying concentration–responses on the basis of efficacy, the number of data points observed above background activity, and the quality of fit. To facilitate analysis, each curve class was combined with an efficacy cutoff and converted to a numeric curve rank such that more potent and efficacious compounds with higher quality curves were assigned a higher rank (see Supplemental Material, Table 4). Curve ranks should be viewed as a numeric measure of compound activity. Compounds that showed activation/inhibition in both the ratio and the 460-nm readouts were defined as activators/inhibitors. Among the activators/inhibitors, compounds with curve rank ≥ 5 or ≤ –5 in the ratio readout were further defined as active activators (agonists)/inhibitors (antagonists). Compounds with curve rank 0 (curve class 4) in both the ratio and 460-nm readouts were defined as inactive, and compounds with other phenotypes were defined as inconclusive. Curve rank, potency, and efficacy data generated on all compounds and assays can be downloaded from Huang (2010).

## Results

An initial evaluation of assay performance showed that the assays behaved as expected in terms of biological activity of known agonists and antagonists included as positive control compounds in the screening libraries. The Tox21 compound collection contains a set of 54 known NR ligands assembled by Sigma (St. Louis, MO) (Sigma 2007). Table 1 lists the ligands with known interactions with NRs included in the present study and their activities observed [phenotype and half-maximal effective concentration (EC_50_) or inhibitory concentration (IC_50_)] in the corresponding NR assays. We positively identified all of these known ligands with our assays, with expected potencies confirming the utility of these NR assays. Some of the apparent actives identified from the LXRβ assays indicated a potential problem with the LXRβ cell line (data not shown). We thus excluded data from these assays from further analysis.

**Table 2:** Compound reproducibility definitions.

Replicate no. 1	Replicate no. 2	Call
Agonist*a*		Agonist		Active match
Antagonist*b*		Antagonist		Active match
Inactive*c*		Inactive		Inactive match
Agonist		Inactive		Mismatch
Inactive		Agonist		Mismatch
Antagonist		Inactive		Mismatch
Inactive		Antagonist		Mismatch
Agonist		Antagonist		Mismatch
Antagonist		Agonist		Mismatch
Other*d*		Other		Inconclusive
**a**Compound with curve rank ≥ 5. **b**Compound with curve rank ≤ –5. **c**Compound with a curve rank of 0. **d**“Other” includes compounds that showed inconclusive activities with nonzero curve ranks between –4 and 4.				

**Figure 1:**
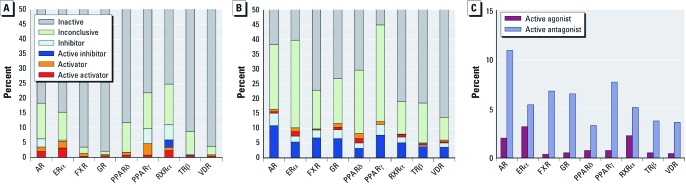
Distributions of compound activity outcomes in the agonist-mode (*A*) and antagonist-mode (*B*) NR assays. (*C*) Distribution of active agonists and antagonists.

Figure 1A,B shows the distributions of compound activity outcomes in the agonist- and antagonist-mode assays, respectively. In general, more compounds were active in the antagonist-mode assays than in the agonist-mode assays (Figure 1C), in which cytotoxicity might be playing a role. The percentage of compounds classified as active ranged from 0.4% (FXR) to 3.2% (ERα) in the agonist-mode assays and from 3.3% (PPARδ) to 10.9% (AR) in the antagonist-mode assays. When we excluded compounds identified as potentially autofluorescent and/or cytotoxic (see below for criteria applied), the fractions of apparent activators and inhibitors decreased for both the agonist- and antagonist-mode assays (data not shown), but the number of apparent active compounds remained larger in the antagonist-mode assays, although by a smaller margin.

**Figure 2:**
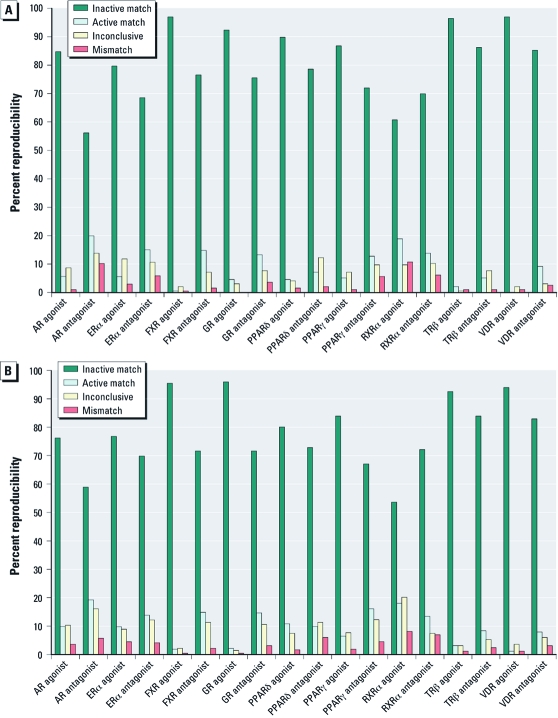
Intralibrary (*A*) and interlibrary (*B*) compound reproducibility across different NR assays. Intralibrary reproducibility is calculated by comparing the activity of copies of each compound replicated within the U.S. EPA or NTP compound library. Interlibrary reproducibility is calculated by comparing the activity of the NTP copy and U.S. EPA copy of each compound presented in both the U.S. EPA and NTP libraries.

*Compound reproducibility.* There were 130 compounds replicated in the U.S. EPA plate and 66 compounds in the NTP plate, and 416 compounds overlapped between the U.S. EPA and the NTP plates. We calculated compound reproducibility for all NR assays in both agonist and antagonist mode using the ratio readout. We first defined each compound replicate as an agonist, an antagonist, inconclusive, or inactive based on its curve rank [for details, see Supplemental Material, Table 4 (doi:10.1289/ehp.1002952)]. We then made three types of reproducibility calls (match, mismatch, and inconclusive) based on the concordance of each replicate (Table 2). Overall, the intraplate replicates showed slightly better reproducibility (88.6%) than did interplate replicates between the U.S. EPA and NTP libraries (85.5%). Both mismatch (4.1%) and inconclusive (10.4%) rates were slightly higher for the interplate than for the intraplate replicates (3.5% mismatch and 7.9% inconclusive). Variations in compound reproducibility were assay dependent (Figure 2). The overall matching rate, counting both intra- and interplate replicates, ranged from 97.7% (GR agonist) to 74.2% (RXRα agonist); mismatch rates ranged from 0.3% and 0.5% (GR and FXR agonist mode) to 9.0% (RXRα agonist), and inconclusive rates ranged from 2.0% (GR agonist) to 16.8% (RXRα agonist).

The reproducibility of an assay is a good indicator of assay performance and quality. To generate a single measure of reproducibility so that all assays could be easily compared, we scored all NR assays (2× % active match + % inactive match – % inconclusive – 2× % mismatch) and ranked them by this score, sorted in descending order (Table 3). We calculated the reproducibility score in a way such that assays with higher concordance rates and lower mismatch rates would be ranked higher. We assigned more weight to active matches and mismatches because we derived these results from more reliable concentration–response curves and to account for the overall low active rate. We assigned each assay an arbitrary grade: A (score, ≥ 90%), B (≥ 80% to 90%), C (≥ 70% to 80%), or D (< 70%), with A being the highest-quality assays in terms of reproducibility and D the lowest. The grades are meant only to serve as a guide for assay prioritization. With this ranking scheme, we ranked the GR and FXR agonist-mode assays as the best-performing assays and the RXRα agonist-mode assay as the worst performing, with the lowest data reproducibility. The agonist-mode assays performed better overall than did the antagonist-mode assays—among the top 50% performing assays, six were agonist-mode assays and only three were antagonist-mode assays. The top five assays were all agonist mode. This outcome was not entirely unexpected because the antagonist-mode assays all required the pre-addition of nonsaturating levels of an agonist compound to stimulate the receptor signal before test compounds could be screened, which introduces an extra source of variance.

**Table 3:** NR assays ranked by their compound reproducibility.

Assay	Mismatch (%)	Inconclusive (%)	Match (%)	Reproducibility score (%)	Assay grade*a*
GR agonist		0.3		2.0		97.7		98.0		A
FXR agonist		0.5		2.1		97.4		95.8		A
TRβ agonist		1.1		2.3		96.6		94.8		A
VDR agonist		1.1		3.1		95.8		91.2		A
PPARδ agonist		1.6		6.4		92.0		91.2		A
TRβ antagonist		2.0		6.0		92.0		89.4		B
VDR antagonist		2.9		5.1		92.0		89.4		B
FXR antagonist		2.0		10.0		88.1		89.1		B
PPARγ agonist		1.6		7.5		90.8		86.1		B
GR antagonist		3.3		9.6		87.1		85.1		B
AR agonist		2.8		9.8		87.4		80.6		B
PPARγ antagonist		4.9		11.4		83.7		77.5		C
ERα agonist		4.0		9.8		86.2		76.9		C
RXRα antagonist		6.7		8.3		85.0		76.8		C
ERα antagonist		4.8		11.5		83.6		76.8		C
PPARδ antagonist		4.7		11.6		83.7		71.6		C
AR antagonist		7.2		15.4		77.5		67.2		D
RXRα agonist		9.0		16.8		74.2		57.7		D
**a**Assays with grade A were considered the highest quality assays in terms of reproducibility, and D the lowest.										

*Using single-channel readouts of* bla *assays to assess autofluorescence and cytotoxicity.* Compound autofluorescence and cytotoxicity can interfere with assay readouts and produce artificial results, because fluorescent compounds could appear as activators and show up as false positives in agonist-mode assays, and cytotoxic compounds could appear as inhibitors because of reduced cell viability and show up as false positives in antagonist-mode assays. The green channel (530-nm readout) is the control channel of the *bla* assay. Increased or decreased fluorescence activity in this channel can be interpreted as an indicator of compound autofluorescence or cytotoxicity (Xia et al. 2009b). Blue fluorescent compounds may not be detected in the 530-nm readout but could still interfere with the 460-nm readout, which is the blue, reporter-gene–dependent signal channel of the *bla* assay. Therefore, pan-activation in the blue channel across multiple NR assays would also indicate compound autofluorescence. We then identified a compound as autofluorescent if it showed activation in > 10 of 20 agonist-mode assay readouts (counting both the 530-nm and 460-nm readouts separately) or if it showed activation in the 460-nm readout in more than four agonist-mode assays and was identified as fluorescent (activity > 10% fluorescent control compound) at 460 nm in the autofluorescence spectra scans (Simeonov et al. 2008). Using these criteria, we identified 25 compounds (11 from NTP and 14 from U.S. EPA) as autofluorescent and excluded them from further analysis [see Supplemental Material, Table 5 (doi:10.1289/ehp.1002952)]. The criteria chosen for identifying autofluorescent compounds (as well as cytotoxic compounds) were empirical and were used to minimize false positives. Most of the compounds identified as autofluorescent by this method were well-known fluorophores, partially validating the approach. Further experimental studies are needed to fully confirm the apparent artifacts.

The cell viability assay has been widely used as a measure of compound cytotoxicity (Xia et al. 2008). We identified active compounds (non-class 4) identified by the cell viability assay in parental HEK 293 cells [for a description of the cell viability assay, see Supplemental Material (doi:10.1289/ehp.1002952)], or compounds that reduced activity in the 530-nm readout of more than four antagonist-mode assays, as cytotoxic and excluded them from further analysis of the antagonist-mode data. We identified a total of 323 compounds as potentially cytotoxic, 152 of which were from the NTP collection and 171 from the U.S. EPA collection (Supplemental Material, Table 6). Cytotoxicity interference is less of a concern for agonist-mode assays than for antagonist-mode assays because agonists that are cytotoxic at higher concentrations generate bell-shaped (inverted-U) concentration–response curves in the 460-nm channel. For more discussion on how activities in the 460-nm and 530-nm reads correlated with the cell viability assay results, see Supplemental Material, “Using single channel readouts of *bla* assays to assess cytotoxicity” and Supplemental Material, Figure 1.

**Figure 3:**
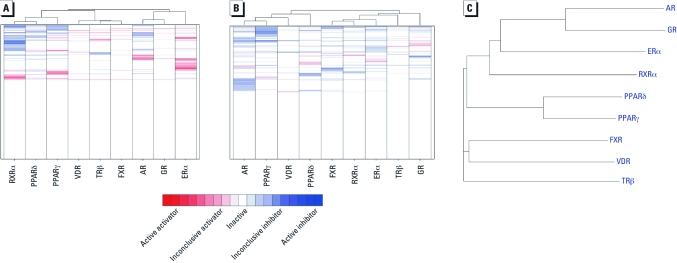
Comparison of the human NR LBD similarity and compound activity-pattern similarity. (*A*) and (*B*) Hierarchical clustering (Spotfire DecisionSite, version 8.2; Spotfire Inc., Cambridge, MA) of the agonist-mode (*A*) and antagonist-mode (*B*) NR assays using the correlation of the compound curve ranks (from the ratio readout) as the similarity metric, where each row represents a compound and each column represents an NR assay. The heat maps are colored based on compound activity: compounds that showed apparent activation (red) and inhibition (blue); less conclusive activators or inhibitors are colored a lighter shade of red or blue; inactive compounds are shown in white. (*C*) Phylogram of the LBDs of the nine human NRs tested. Amino acid sequences of the LBDs were downloaded from the PubMed protein database (PubMed 2010) and aligned using ClustalW2 (European Molecular Biology Laboratory–European Bioinformatics Institute 2009).

*Chemical genomics: compound activity-pattern similarity and NR LBD sequence homology.* The agonist-mode and the antagonist-mode NR assays were both hierarchically clustered using the correlation of the compound curve ranks (from the ratio readout) as the similarity metric. We excluded compounds identified as potentially autofluorescent from the clustering exercises and also excluded compounds identified as potentially cytotoxic from the clustering of the antagonist-mode assays. Figure 3A,B shows results for agonist and antagonist mode, respectively. The agonist-data–based clustering of NRs (Figure 3A) matched nearly perfectly with the NR LBD sequence homology [Figure 3C; see also Supplemental Material, Figure 2 (doi:10.1289/ehp.1002952)] (Laudet 1997; Zhang et al. 2004), where the agonist data clustering segregated the nine NRs into two major branches (Figure 3A): the ER-like branch, with ERα, AR, and GR, and the thyroid hormone receptor (TR)-like branch, with RXRα and the rest of the NRs. The clustering again further divided the TR-like branch into two subgroups, one containing TRβ, FXR, and VDR and the other containing the two PPARs and RXRα. The only difference from the sequence clustering (Figure 3C) was that PPARδ clustered more closely with RXRα than with PPARγ in the assay-data–based clustering (Figure 3A).

The antagonist-mode data–based clustering of the NRs (Figure 3B) showed poor concordance with the NR LBD sequence homology (Figure 3C). The ER-like subfamily members were segregated into two different branches, with ERα and GR in one branch and AR clustered into the other branch (Figure 3B). This was surprising because GR is more closely related to AR than to ERα in terms of sequence similarity (Figure 3C). The clustering grouped the TR-like subfamily into two clusters as well, with VDR, PPARδ, and PPARγ in one cluster and TRβ and FXR in the other (Figure 3B). The two PPARs are the most closely related by sequence (Figure 3C), but this was not reflected in the antagonist-mode–based phenotype clustering (Figure 3B).

**Figure 4:**
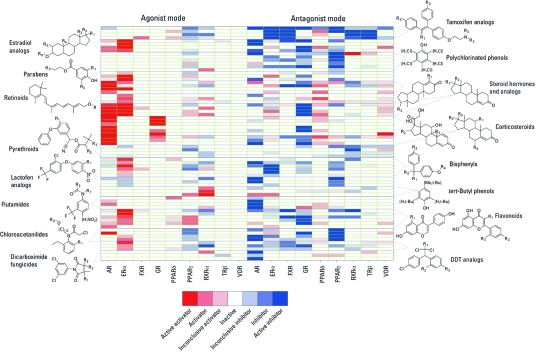
Example structure classes with consistent NR activity patterns or signatures. Compounds were clustered by structure similarity using the SOM algorithm. Compounds in the same cluster belong to the same structure class. The structure classes shown contain compounds with similar activity patterns as well. DDT, dichlorodiphenyltrichloroethane.

*SAR analysis.* We clustered all compounds in the NTP and U.S. EPA libraries based on structural similarity (2,048-bit Daylight fingerprints; Daylight Chemical Information Systems, Inc., Laguna Niguel, CA) using the self-organizing map (SOM) algorithm (Kohonen 2006), yielding 336 clusters. Despite of the diversity in response [for details, see Supplemental Material, “Structural diversity assessment of apparent NR agonists and antagonists,” and Supplemental Material, Figure 3 (doi:10.1289/ehp.1002952)], we identified 16 classes of compounds with consistent NR activity patterns by examining the activity patterns of compounds within each structure cluster. Figure 4 shows the structure scaffolds and NR activities of these compound classes. Among these are many known ligands or disruptors of NRs whose activities observed in our study was consistent with their known NR activities. Examples include the known ERα-active classes of compounds, such as the estradiol and tamoxifen analogs, parabens (Harvey and Everett 2004), bisphenyls (including bisphenol A, bisphenol B, and methoxychlor), and flavonoids; steroid hormones and analogs and flutamides with known AR activity; and corticosteroids with known GR activity. We also observed that subtle changes in structures of compounds belonging to the same class led to variations in their NR activity. For example, the class of steroid hormones was clustered into several subclasses based on their NR activity patterns, where the sex hormones appeared as agonists of AR and ERα and the corticosteroids showed activities mostly against AR and GR. Another example is the ERα activity of the flavonoids, where the isoflavones (e.g., genistein) were identified as more conclusive/potent ERα agonists than the normal flavonoids (e.g., kaempferol). Several classes of compounds, including the lactofen analogs (Butler et al. 1988) and dicarboximide fungicides (Kelce et al. 1994), have been reported to induce liver toxicity. The lactofen analogs appeared primarily as PPARγ and AR antagonists, and the dicarboximide fungicides as AR antagonists, consistent with literature reports (Gray et al. 2001); however, the AR activity of the lactofen analogs has not been reported before. Of the chloroacetanilide herbicides, alachlor (Klotz et al. 1996), and acetochlor (Rollerova et al. 2000) have been reported to have weak estrogenic effects, consistent with their weak activities observed in our ERα assays. However, we consistently identified this class of compounds as PPARγ antagonists in our NR assays as well. The NR activity of these compounds may be related to their liver toxicity.

## Discussion

As Tox21 is moving from the pilot phase to the full production phase where a library of > 10,000 compounds will be screened initially against suites of NRs and stress response pathways, selection of appropriate assays to be included is critical. A set of criteria needs to be established to determine the value and quality of an assay for inclusion into the Tox21 production phase. One approach is to use the existing data from the pilot-phase screens. Taking this approach, we have examined the NR profiling data generated from the pilot phase Tox21 collection of approximately 3,000 environmental chemicals in terms of data reliability, which can be measured by the level of variability/noise, dynamic range, and reproducibility, to assess potential challenges and propose strategies for data-driven assay prioritization. Having replicated compounds in the compound library was useful for assessing assay reproducibility. Assays ranked high on the reproducibility scale (Table 3) could be prioritized for the scaled-up screening of additional compound libraries. For assays that failed the data quality control criteria, which include the grade D assays and possibly the grade C assays listed in Table 3, replacement assays that measure the same biology (same NR in this case) need to be selected with alternative cell types, assay technologies (e.g., luciferase instead of *bla*), or readouts (e.g., luminescence instead of fluorescence).

Unlike the agonist-mode assays, in which the activities observed were nearly a perfect reflection of the NR LBD sequence phylogeny, the antagonist-mode phenotype clustering showed low concordance with sequence homology. One potential cause for this low concordance is that antagonists need not bind as perfectly as do agonists to the LBD to antagonize the receptor activity (Brzozowski et al. 1997; Shiau et al. 1998). Another possible explanation is that a compound may antagonize an NR via allosteric or noncompetitive effects instead of directly binding to the LBD of the NR (Jones et al. 2009), such that the LBD sequence would not contribute to the antagonist phenotype in these NR assays. Finally, cytotoxicity interference, not completely removed to avoid losing true antagonists, could be a playing a role. For example, tamoxifen, a well-known antagonist of the estrogen receptor, is also cytotoxic (Cleator et al. 2009; Dhingra 1999). In contrast, the agonist phenotype clustering of NRs showed good concordance with the sequence clustering results because cytotoxicity interference was minimal in the agonist (gain of signal) mode assays and binding to the LBD is probably necessary for a compound to activate an NR. These are further supported by the higher structure diversity observed for the apparent antagonists than the agonists. The lesson learned from this exercise is that we can use phenotype/chemical response data to infer LBD protein sequence/target relationships to a certain degree. The level of confidence depends on the noise level in the phenotype data. In the case of NRs, the agonist-mode data were more robust than the antagonist-mode data in inferring sequence relationships.

Regardless of the potential interference from compound autofluorescence and cytotoxicity, we showed these assays to be biologically relevant by positively identifying a set of known NR ligands built into the compound collection (Table 1). In addition, SAR analysis revealed a number of structural classes with consistent activity patterns (Figure 4). These classes of compounds not only contain analogs of known NR ligands that further validated the utility of these assays, but also showed NR interactions not previously reported that warrant further toxicological evaluation because the SAR strengthens the validity of the observed activities. Several of these compound classes are known liver toxicants, the toxicity of which could be linked to their endocrine-disrupting potential, and as such, their NR activity patterns could serve as predictive signatures for their toxicity end point (Judson et al. 2010).

## Conclusions

Our analyses of the NR profiling data indicate that this is a valuable data set for generating hypotheses and establishing metrics for assay prioritization, and that we can strategically identify and reduce interpretive interference from assay artifacts. Moreover, LBD sequence and compound SAR analyses provide support to the utility of these qHTS assays in their ability to identify known endocrine-disrupting environmental chemicals and revealed classes of chemicals with activity patterns that could serve as bases for in-depth toxicological testing prioritization and provide clues for toxicity mechanism interpretation.

## Supplemental Material

(440 KB) PDF
