# Supplementary material for: Chemical Genomics Profiling of Environmental Chemical Modulation of Human Nuclear Receptors
Source: Environ Health Perspect. 2011 May 4;119(8):1142–8. doi: 10.1289/ehp.1002952 (PMC3237348; doi:10.1289/ehp.1002952)
Supplement: (440 KB) PDF [file ehp.1002952.s001.pdf]

Supplemental Material

Chemical Genomics Profiling of Environmental Chemical Modulation of Human Nuclear Receptors

Ruili Huang, Menghang Xia, Ming-Hsuang Cho, Srilatha Sakamuru, Paul Shinn, Keith A. Houck, David J. Dix, Richard S. Judson, Kristine L. Witt, Robert J. Kavlock, Raymond R. Tice, Christopher P. Austin

Table of Contents

Compound Collection 3

β-Lactamase reporter gene assay and qHTS 3

Cell lines and culture conditions 3

qHTS Data Analysis 7

Using single channel readouts of bla assays to assess cytotoxicity 10

Cell viability assay 10

Structural diversity assessment of apparent NR agonists and antagonists 13

References 25

Tables

Table 1. Antibiotics used in cell culture medium for each nuclear receptor cell line 4

Table 2. Plate statistics as a measure of assay performance and positive control compounds used for each nuclear receptor assay 6

Table 3. Amended qHTS curve classification 8

Table 4. Definition of curve rank as a numeric measure of compound activity 9

Table 5. Compounds in the Tox21 collection that were identified as potentially auto fluorescent 10

Table 6. Compounds in the Tox21 collection that were identified as potentially cytotoxic 17

Figures

Figure 1. Correlations of curve ranks between the cell viability data and the 530 nm and 460 nm reads of each NR assay 12

Figure 2. Phylogenetic tree of human nuclear receptors based on LBD homology. The ten receptors tested in this study are highlighted in orange. 13

Figure 3. Structure diversity of the agonists (a) and antagonists (b) for each NR 15

Compound Collection

The current Tox21 compound collection consists of 2870 compounds, 1408 of which were provided by NTP (Xia et al. 2008) and 1462 provided by the EPA (Huang et al. 2009; Judson et al. 2009). There were 130 compounds replicated in the EPA collection, 66 compounds in the NTP collection, and an overlap of 416 compounds between the EPA and the NTP collections (different salt forms of the same parent molecule were considered as replicates). These replicated compounds, determined based on canonical SMILES, were usually (but not always) from different lots and sources. The structures of these compounds are available at PubChem (PubChem 2007, 2009). The compounds were dissolved in DMSO either as 10 mM (NTP) or 20 mM (EPA) stock solutions. Compound plates with 14 concentrations ranging from 0.5 nM to 92 μM were prepared in 1536-well plate format (Xia et al. 2008). During screening, the 1536-well compound plates were stored at room temperature and sealed when not in use. The other copies of 1536-well compound plates were maintained at -80 °C for long-term storage.

β-Lactamase reporter gene assay and qHTS

Cell lines and culture conditions GeneBLAzer® AR-UAS-*bla* GripTite™ cell line, GeneBLAzer® ERα-UAS-*bla* GripTite™ cell line, GeneBLAzer® FXR-UAS-*bla* HEK 293T cell line, CellSensor® GR-MMTV-*bla* HeLa cell line, GeneBLAzer® LXRβ-UAS-*bla* HEK 293T cell line, GeneBLAzer® PPARδ-UAS-*bla* HEK 293T cell line, GeneBLAzer® PPARγ -UAS-*bla* HEK 293H cell line, GeneBLAzer® RXRα-UAS-*bla* HEK 293T cell line, GeneBLAzer® TRβ-UAS-*bla* HEK 293T cell line, and

GeneBLAzer® VDR-UAS-*bla* HEK 293T cell line were obtained from Invitrogen. These cell lines constitutively co-express a fusion protein comprised of the ligand-binding domains (LBD) of related human nuclear receptors coupled to the DNA-binding domain of the yeast transcription factor GAL4. When activated, these fusion proteins then stimulate β-lactamase reporter gene expression. All the cell lines were cultured in DMEM or DMEM/GlutaMAX™ supplemented with 10% dialyzed fetal bovine serum, 0.1 mM non-essential amino acids, 1 mM sodium pyruvate, 25 mM HEPES, 50 U/mL penicillin and 50 µg/mL streptomycin, and selection antibiotics. Specific antibiotics used in cell culture medium for each cell line are listed in Supplemental Table 1. All cell culture reagents were obtained from Invitrogen. The cultures were maintained in a 37°C incubator with 5% CO_2 and under a humidified atmosphere.

Supplemental Material, Table 1. Antibiotics used in cell culture medium for each nuclear receptor cell line.

Receptor name	Hygromycin (µg/ml)	Zeocin (µg/ml)	Blasticidin (µg/ml)	Geneticin (µg/ml)
AR	80	80	-	-
ERα	80	100	-	-
FXR	100	100	-	-
GR	-	-	5	-
LXRß	80	-	-	-
PPARδ	80	100	-	-
PPARγ	100	-	-	500
RXRα	100	100	-	-
TRß	80	100	-	-
VDR	80	80	-	-

β-Lactamase reporter gene assay and qHTS Compound formatting and qHTS were performed as described previously (Xia et al. 2009). Briefly, the nuclear receptor related *bla* cells were suspended either in phenol red-free DMEM medium supplemented with

2% charcoal/dextran stripped fetal bovine serum (FBS) or OPTI-MEM medium supplemented with 2% dialyzed FBS. The assay medium also contains 0.1 mM nonessential amino acids, 1 mM sodium pyruvate, 100 U/mL penicillin, and 100 μg/mL streptomycin. The cells were dispensed at 1,500 to 5,000 cells/5 (for antagonist mode) or 6 (for agonist mode) μL/well in 1,536-well black wall/clear bottom plates (Greiner Bio-One North America, NC) using a Flying Reagent Dispenser (FRD) (Aurora Discovery, CA) during assay optimization or Thermo Scientific Multidrop Combi (Thermo Fisher Scientific Inc., Waltham, MA) for screening. After the cells were incubated at 37°C for 5 to 6 h to allow for cell attachment, 23 nL of compounds at 14 or 15 concentrations from the NTP and EPA compound collections was transferred to the assay plate by a pin tool (Kalypsys, San Diego, CA). For antagonist mode, 1 μL assay medium either with or without the known agonist for each nuclear receptor was added into assay plates using a FRD. Positive control compounds and their concentrations used for each assay are listed in Supplementary Table 2. For agonist mode assays, positive control compounds were dispensed into one of the first four columns on each plate. For antagonist mode assays, positive control compounds were dispensed into every well on each plate, except for one of the first four columns, where only DMSO was dispensed. The final concentration of the compounds in the 6 μL assay volume ranged from 0.5 nM to 92 μM. The plates were incubated for 16 to 18 h at 37 °C depending on the particular NR cell line. One μL of LiveBLAzer™ B/G FRET substrate (Invitrogen, Carlsbad, CA) detection mix was added and the plates incubated at room temperature for 1.5 to 2 h. Fluorescence intensity (405 nm excitation, 460 and 530 nm emission) was measured using an Envision plate reader (Perkin Elmer, Shelton, CT). Data was expressed as the ratio of 460 nm/530 nm

emissions. The plate statistics (signal to background (S/B) ratios, Z's, CVs) indicating assay performance are also listed in Supplementary Table 2. All assays had Z′ factors >0.4 and 17 out of 20 assays had Z′ >0.5. All 20 assays had S/B ratios ≥2, 14 out of 20 assays had CV<10 and 17 assays had CV<15. Less than ideal CVs (>10) in agonist mode assays generally reflect low basal level signals, and are not of concern if the S/B ratios are high because gain of signal is measured. However, for antagonist mode assays (where loss of signal is measured), this could generate larger variations in the data.

Supplemental Material, Table 2. Plate statistics as a measure of assay performance and positive control compounds used for each nuclear receptor assay

Assay	S/B[a]	Z'[b]	CV[c]	Positive Control (μM)	Control Titration EC50/IC50 (μM)
AR agonist	2.2±0.2	0.60±0.10	10±2	R1881 (0.03)	<0.001
ERα agonist	2.5±0.5	0.64±0.14	11±4	17β-estradiol (0.02)	0.02±0.03
FXR agonist	5.4±0.5	0.68±0.08	9±4	Chenodeoxycholic acid (60)	29±5
GR agonist	4.2±0.5	0.59±0.11	16±2	Dexamethasone (0.1)	0.001±0.0002
LXR agonist	3.0±0.2	0.76±0.11	9±4	T0901317 (15)	1.2±0.5
PPARδ agonist	2.4±2.5	0.75±0.04	9±3	L165041 (10)	0.036±0.006
PPARγ agonist	3.4±0.2	0.89±0.02	7±2	Rosiglitazone (2)	0.005±0.001
RXRα agonist	1.9±0.1	0.74±0.05	8±1	Retinoic acid (0.5)	0.0002±0.0002
TRβ agonist	2.8±0.5	0.51±0.19	16±2	T3 (0.01)	0.001±0.005
VDR agonist	4.3±0.5	0.83±0.05	7±1	1α,25-Dihydroxy vitaminD3 (0.05)	0.001±0.0003
AR antagonist	2.5±0.8	0.65±0.07	15±1	R1881 (0.01)	2.7±0.7 (Cyp Ac)
ERα antagonist	2.7±1.1	0.43±0.08	9±0.4	17β-estradiol (0.0005)	0.07±0.07 (4-Hydroxytamoxifen)
FXR antagonist	4.9±0.4	0.73±0.05	7±1	Chenodeoxycholic acid (50)	34±5 (Guggulsterone)
GR antagonist	2.8±0.2	0.49±0.07	10±1	Dexamethasone (0.002)	0.02±0.01 (Mifepristone)
LXR antagonist	3.5±0.6	0.62±0.38	12±3	T0901317 (1.5)	N/A
PPARδ antagonist	1.8±0.1	0.49±0.06	6±1	L165041 (0.5)	54±16 (MK886)
PPARγ antagonist	2.7±0.2	0.84±0.07	3±0.3	Rosiglitazone (0.05)	0.011±0.002 (GW9662)
RXRα antagonist	2.1±0.2	0.78±0.07	8±3	Retinoic acid (0.1)	N/A
TRβ antagonist	3.0±0.5	0.55±0.11	7±1	T3 (0.0004)	N/A
VDR antagonist	4.2±0.3	0.87±0.03	4±1	1α,25-Dihydroxy vitaminD3 (0.003)	N/A

[a] Signal-to-background ratio. S/B = median positive control signal / median neutral (DMSO) control signal

[b] Z' = 1 - (3SD of positive control + 3SD of neutral control) / (median of positive control – median of neutral control)

[c] Coefficient of variation of signals from the sample region of the plates. CV = SD of sample region / median of sample region

qHTS Data Analysis

Analysis of compound concentration–response data was performed as previously described (Inglese et al. 2006). Briefly, raw plate reads for each titration point were first normalized relative to the positive control compound (agonist mode: 100%; antagonist mode: 0%) (control compounds and concentrations used can be found in Supplementary Table 2) and DMSO-only wells (agonist mode: 0%; antagonist mode: -100%) as follows: % Activity = $((V_{compound} - V_{DMSO})/(V_{pos} - V_{DMSO})) \times 100$, where $V_{compound}$ denotes the compound well values, V_{pos} denotes the median value of the positive control wells, and V_{DMSO} denotes the median values of the DMSO-only wells, and then corrected by applying a NCGC in house pattern correction algorithm using compound-free control plates (i.e., DMSO-only plates) at the beginning and end of the compound plate stack. Concentration–response titration points for each compound were fitted to a four-parameter Hill equation (Hill 1910) yielding concentrations of half-maximal activity (AC_{50}) and maximal response (efficacy) values. Compounds were designated as Class 1–4 according to the type of concentration–response curve observed (Inglese et al. 2006). Curve classes are heuristic measures of data confidence, classifying concentration–responses on the basis of efficacy, the number of data points observed above background activity, and the quality of fit. The qHTS curve classification scheme has been recently amended to better suit the needs of toxicology research (Supplementary Table 3). Compounds that showed activation/inhibition in both the ratio and the 460 nm readouts

were defined as activators/inhibitors. Among the activators/inhibitors, compounds with class 1.1, 1.2, 2.1 or 2.2 curves (p<0.05) and >60% efficacy in the ratio readout were further defined as active activators (agonists)/inhibitors (antagonists). Compounds that were class 4 in both the ratio and 460 nm readouts were defined as inactive and compounds with other phenotypes were defined as inconclusive. To facilitate analysis, each curve class was combined with an efficacy cutoff and converted to a numerical curve rank such that more potent and efficacious compounds with higher quality curves were assigned a higher rank, and inactive (class 4) compounds were assigned curve rank 0. A detailed definition of curve rank is shown in Supplementary Table 4. Curve ranks should be viewed as a numerical measure of compound activity.

Supplemental Material, Table 3. Amended qHTS curve classification

Curve Class	Description	Efficacy	*p*-value*	Asymptotes	Inflection
1.1	Complete curve	>6SD†	<0.05	2	Yes
1.2	Complete curve	≤6SD; >3SD	<0.05	2	Yes
1.3	Complete curve	>6SD	≥0.05	2	Yes
1.4	Complete curve	≤6SD; >3SD	≥0.05	2	Yes
2.1	Incomplete curve	>6SD	<0.05	1	Yes
2.2	Incomplete curve	≤6SD; >3SD	<0.05	1	Yes
2.3	Incomplete curve	>6SD	≥0.05	1	Yes
2.4	Incomplete curve	≤6SD; >3SD	≥0.05	1	Yes
3	Single point activity	>3SD	NA	1	No
4	Inactive	≤3SD	≥0.05	0	No
5‡	Inconclusive	NA	NA	NA	NA

* *p*-value is derived from a F-test that measures the quality of curve fit.
† SD is the standard deviation of sample activities at the lowest tested concentration and values of the DMSO control wells.
‡ Class 5 is a special class for samples with activity at zero concentration (zero activity; extrapolated) exceeding 6SD or with zero activity>3SD and the difference between the maximal change in activity observed in the tested concentration range and zero activity is <3SD.

Supplemental Material, Table 4. Definition of curve rank as a numeric measure of compound activity

Curve class	Efficacy	Curve rank	Activity Category
1.1		9	agonist
1.2	>50%	8	agonist
2.1		7	agonist
1.2	≤50%	6	agonist
2.2	>50%	5	agonist
2.2	≤50%	4	inconclusive
1.3		3	inconclusive
1.4		3	inconclusive
2.3		2	inconclusive
2.4		2	inconclusive
3		2	inconclusive
5		1	inconclusive
4		0	inactive
-2.3		-2	inconclusive
-2.4		-2	inconclusive
-3		-2	inconclusive
-1.3		-3	inconclusive
-1.4		-3	inconclusive
-2.2	≤50%	-4	inconclusive
-2.2	>50%	-5	antagonist
-1.2	≤50%	-6	antagonist
-2.1		-7	antagonist
-1.2	>50%	-8	antagonist
-1.1		-9	antagonist

Supplemental Material, Table 5. Compounds in the Tox21 collection that were identified as potentially auto fluorescent

Sample ID	CAS	Name
AB02540564-01	458-37-7	Curcumin
AB07930266-01	207-08-9	Benzo(k)fluoranthene
AB07935027-01	396-01-0	Triamterene
AB07935028-01	205-99-2	Benzo(b)fluoranthene
AB07944059-01	65558-69-2	1,3-Diiminobenz (f)-isoindoline
AB07944725-01	22888-70-6	Silibinin
AB07949348-01	130-17-6	2-(4-Aminophenyl)-6-methylbenzothiazole sulfonic acid
AB08080895-01	613-13-8	2-Aminoanthracene
AB08080899-01	91-44-1	7-Diethylamino-4-methylcoumarin
AB08080928-01	2107-76-8	5,7-Dihydroxy-4-methylcoumarin
AB08582940-01	52417-22-8	9-Aminoacridine HCl H2O
NCGC00022393-05	61-68-7	Mefenamic acid
NCGC00022570-07	88107-10-2	LY 171883
NCGC00023458-07	396-01-0	Triamterene
NCGC00023462-04	479-13-0	coumestrol
NCGC00024135-11	53-86-1	Indomethacin
NCGC00090792-04	91-53-2	Ethoxyquin
NCGC00090832-02	207-08-9	Benzo(k)fluoranthene
NCGC00090866-02	205-99-2	Benzo(b)fluoranthene
NCGC00091518-02	57-97-6	7,12-Dimethylbenzanthracene
NCGC00093553-06	54-62-6	Aminofolic acid, 4-
NCGC00094842-05	480-40-0	chrysin
NCGC00162403-02	303-47-9	Ochratoxin A
NCGC00163974-01	191-24-2	Benzo[g,h,i]perylene
NCGC00164420-01	875326-27-5	DRF 2519

Using single channel readouts of bla assays to assess cytotoxicity

Cell viability assay Cell viability after compound treatment was measured using a luciferase-coupled ATP quantitation assay (CellTiter-Glo viability assay, Promega, Madison, WI) in parental HEK293 cells. Total intracellular ATP content corresponds to the number of metabolically competent cells after compound treatment. The cells were dispensed at 2,000 cells/5 μL/well in 1,536-well white/solid bottom assay plates using an FRD. The cells were incubated a minimum of 5 h at 37°C, followed by the addition of

compounds using the pin tool. The assay plates were incubated for 18 h at 37°C, followed by the addition of 5 μL/well of CellTiter-Glo reagent. After 30 min incubation at room temperature, the luminescence intensity of the plates was measured using a ViewLux plate reader (PerkinElmer).

The HEK293 viability data was used as a standard measure of cytotoxicity to assess how the activities in the 530 nm and 460 nm reads correlate with compound cytotoxicity. Correlations of curve ranks (see Supplementary Information for definition) between the cell viability data and the 530 nm and 460 nm reads of each NR assay are shown in Supplementary Figure 1. Inhibition in the 460 nm read of the antagonist mode assays showed the best correlation with cytotoxicity with R averaging around 0.4. Since the 460 nm channel is the reporter gene signal channel, this observation suggests that inhibition seen in this channel could be indicative of cytotoxicity instead of true antagonism of the receptor. The 530 nm read of the antagonist mode assays showed good correlations with the cell viability data as well; however, all except two of the nine NR assays showed negative correlations with viability, that is, activation rather than inhibition of the 530 nm channel is indicative of cytotoxicity for these assays. The 530 nm and 460 nm reads of the agonist mode assays did not show strong correlations with cell viability except for a few cases.

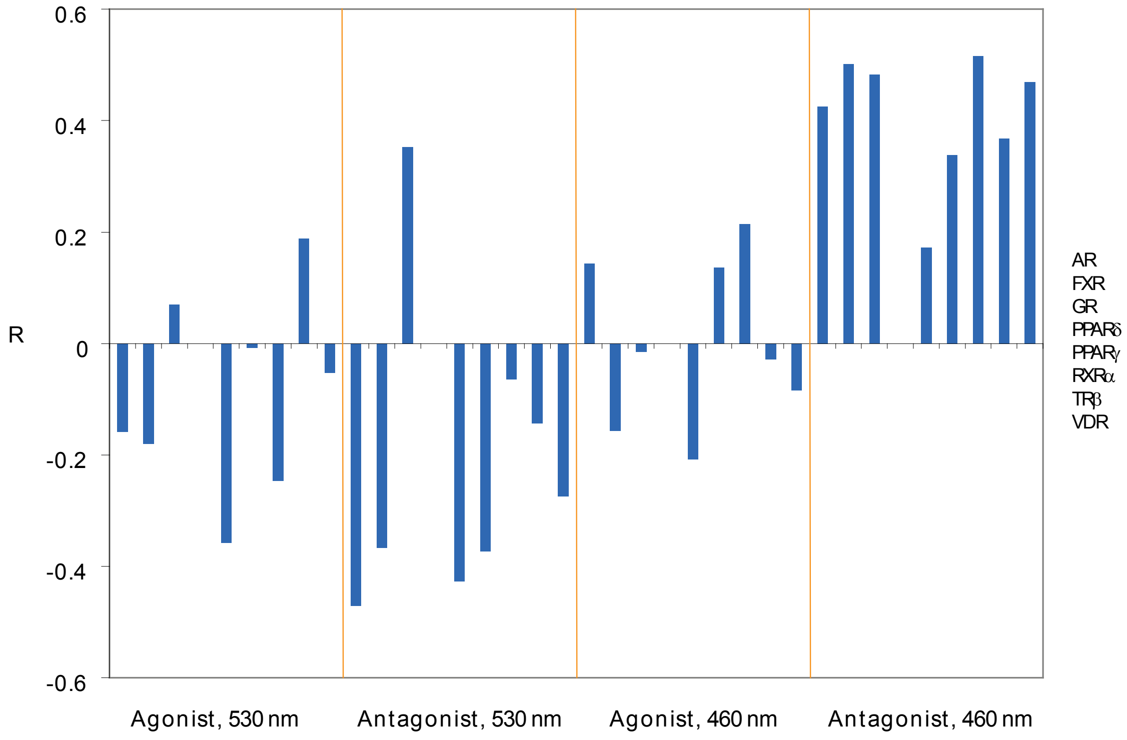

Supplemental Material, Figure 1. Correlations of curve ranks between the cell viability data and the 530 nm and 460 nm reads of each NR assay.

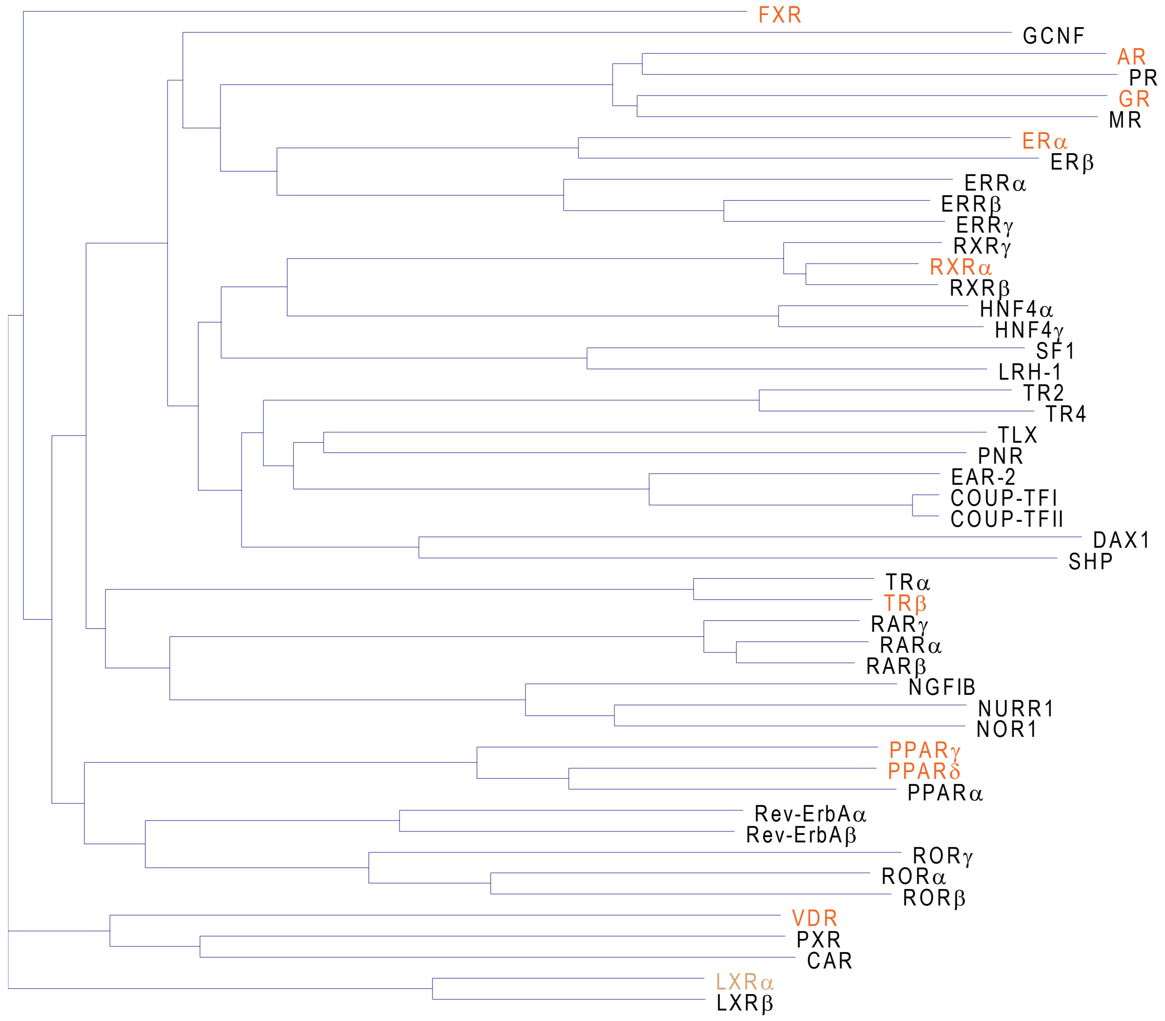

Supplemental Material, Figure 2. Phylogenetic tree of human nuclear receptors based on LBD homology. The ten receptors tested in this study are highlighted in orange.

Structural diversity assessment of apparent NR agonists and antagonists

We assessed the structure diversity of the agonists/antagonists identified for each NR using a "diversity index". Compound structures were first converted into 2048-bit Daylight® fingerprints. The "diversity index" is then defined as the coverage of the structural feature space, represented by the 2048 bits of the Daylight® fingerprint, by these compounds. To account for the effect of sample size (the number of active

compounds for each NR is different, and that a larger number of compounds generally have a better coverage of the structural space, which does not necessarily imply a higher level of structural diversity), a learning curve analysis was performed. Briefly, compounds were selected randomly from the active list of each NR and the fraction of the 2048 features covered by these compounds was calculated. The number of compounds selected for each list ranged from 10 to the size of the list. Each selection was randomized 100 times and the average coverage rate was calculated.

The results for up to 50 compounds selected per NR are shown in Supplementary Figure 3. The diversity of these agonists/antagonists is reflected by the high coverage rates – up to 70-80% of the structure space are covered by 50 compounds from an NR (Supplementary Figure 3) and 100 agonists/antagonists of an NR can cover up to 80-90% of the space (data not shown). The NR antagonists appeared to be more structurally diverse with coverage rates around 40% with 10 compounds and 80% with 50 compounds (Supplementary Figure 3(b)) than the agonists, which showed coverage rates of 25-30% with 10 compounds and 60-70% with 50 compounds (Supplementary Figure 3(a)). Among the agonist mode assays, the TRβ, VDR and PPARδ agonists are the least diverse structurally with coverage rates around 25% with 10 compounds, and the agonists of FXR are the most structurally diverse with 36% coverage followed by PPARγ with coverage rate of 32%, respectively. With 50 compounds, the PPARγ and RXRα agonists came out on top as the most diverse with a coverage rate of 69%. The antagonist mode assays were more uniformly diverse with the 10-compound coverage rate ranging from 43% (ERα) to 33% (PPARδ). Because active compounds from the screening campaigns

were not confirmed by additional assays, false positives were likely present in the compounds analyzed for diversity. The rate of false positives in these assays is currently not known although the reproducibility of the replicated compounds gives some idea. If the cause of the false positive is due to statistical variation or other effects not directly related to binding to the ligand binding domain of the receptor, diversity may be inflated relative to actual selectivity of a specific receptor.

(a)

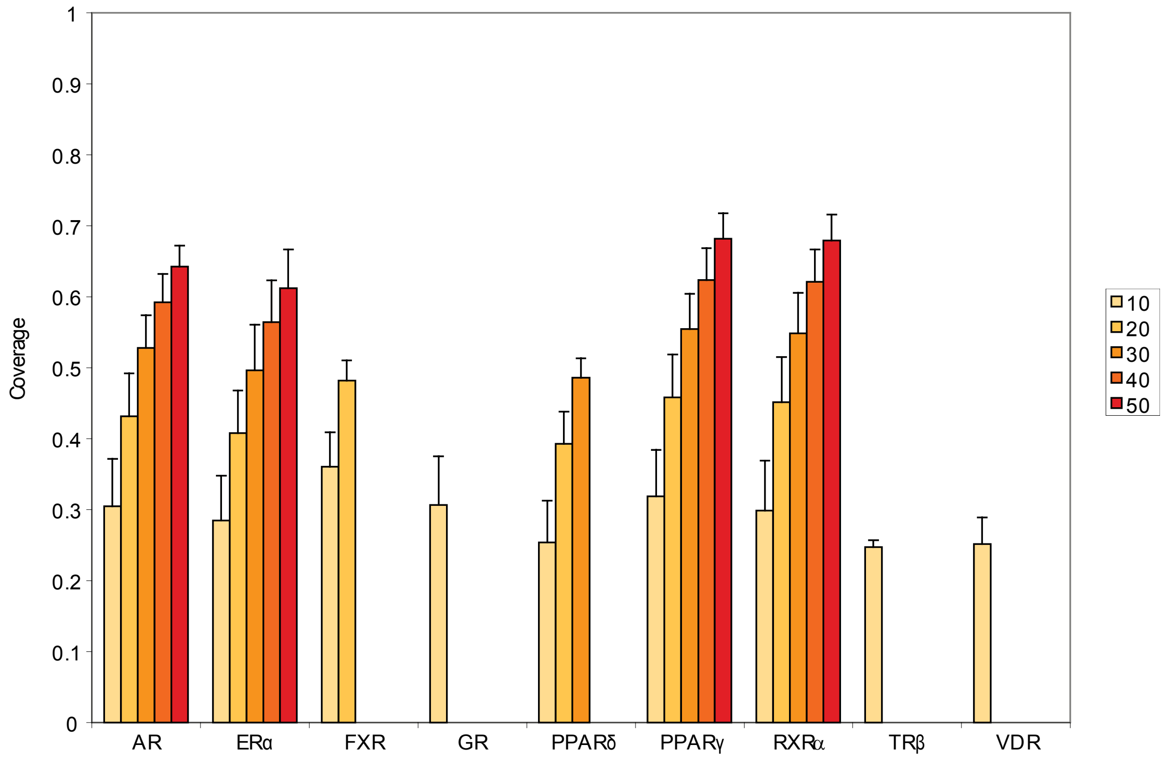

(b)

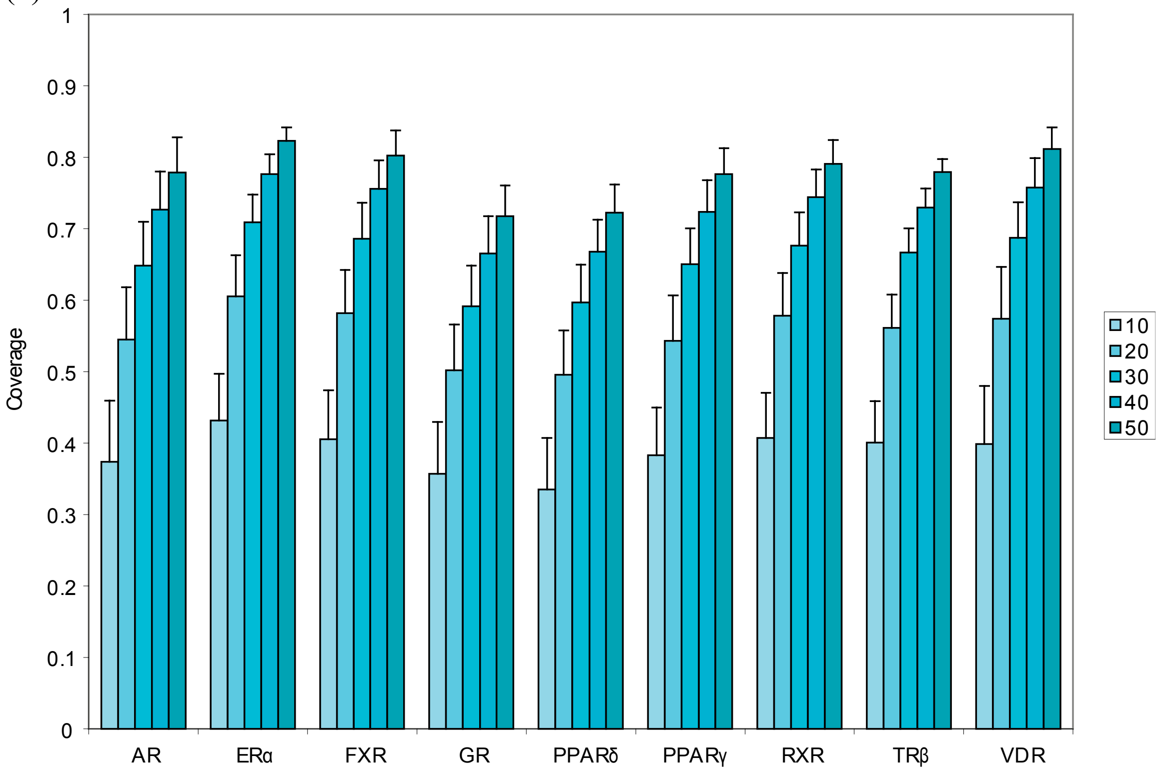

Supplemental Material, Figure 3. Structure diversity of the agonists (a) and antagonists (b) for each NR as measured by the "diversity index", defined as the coverage of the structural feature space. The results for up to 50 compounds selected per NR are shown.

Supplemental Material, Table 6. Compounds in the Tox21 collection that were identified as potentially cytotoxic

Sample ID	CAS	Name
AB02509332-01	25152-84-5	2,4-Decadienal
AB02540517-01	65646-68-6	4-Hydroxyphenyl retinamide
AB02540519-01	7789-12-0	Sodium dichromate dihydrate (VI)
AB02540527-01	7778-50-9	Potassium dichromate
AB02540531-01	148-82-3	Melphalan
AB02540564-01	458-37-7	Curcumin
AB02540565-01	54965-24-1	Tamoxifen, citrate salt
AB02540575-01	55981-09-4	Nitazoxanide
AB02540576-01	7220-79-3	Methylene blue trihydrate
AB02540577-01	25316-40-9	Adriamycin, hydrochloride
AB02540601-01	159989-65-8	Nelfinavir mesylate
AB02540602-01	8003-22-3	D & C Yellow II
AB02911410-01	91-53-2	Ethoxyquin
AB02914401-01	50-76-0	Actinomycin D
AB07859937-01	20830-75-5	Digoxin
AB07930213-01	57-83-0	Progesterone
AB07930229-01	17924-92-4	Zearalenone
AB07930243-01	97-18-7	2,2'-Thiobis(4,6-dichlorophenol)
AB07930266-01	207-08-9	Benzo(k)fluoranthene
AB07930274-01	446-86-6	Azathioprine
AB07930285-01	2243-76-7	Alizarin Yellow R, sodium salt
AB07930286-01	520-36-5	Apigenin
AB07930292-01	115-09-3	Methyl mercuric (II) chloride
AB07930298-01	320-67-2	5-Azacytidine
AB07935027-01	396-01-0	Triamterene
AB07935028-01	205-99-2	Benzo(b)fluoranthene
AB07935479-01	24169-02-6	Econazole nitrate
AB07935653-01	154-42-7	6-Thioguanine (6-TG)
AB07935671-01	130-26-7	Iodochlorohydroxyquinoline
AB07935672-01	123-31-9	Hydroquinone
AB07935832-01	95-54-5	o-Phenylenediamine
AB07942141-01	5436-43-1	2,2',4,4'-Tetrabromodiphenyl Ether (PBDE 47)
AB07944034-01	104-40-5	p -n -Nonylphenol
AB07944048-01	10108-64-2	Cadmium II chloride
AB07944059-01	65558-69-2	1,3-Diiminobenz (f)-isoindoline
AB07944095-01	102-36-3	3,4-Dichlorophenyl isocyanate
AB07944128-01	66-81-9	Cycloheximide
AB07944329-01	143-50-0	Kepone
AB07944331-01	538-71-6	Domiphen bromide
AB07944366-01	14866-33-2	tetra-N-Octylammonium bromide
AB07944368-01	57-83-0	Progesterone
AB07944378-01	55-56-1	Chlorhexidine
AB07944388-01	68047-06-3	4-Hydroxytamoxifen

Table 6, continued

Sample ID	CAS	Name
AB07944392-01	17831-71-9	Tetraethylene glycol diacrylate
AB07944402-01	427-51-0	Cyproterone acetate
AB07944404-01	133-06-2	Captan 90-concentrate (solid)
AB07944411-01	789-02-6	o,p' -DDT
AB07944416-01	63-05-8	4-Androstenedione
AB07944715-01	62-38-4	Phenyl mercuric acetate
AB07944716-01	13463-41-7	Zinc pyrithione
AB07944719-01	66-81-9	Cycloheximide
AB07944721-01	106-51-4	p-Quinone
AB07944738-01	1191-41-9	Ethyl linolenate
AB07944740-01	50-29-3	p,p'-DDT (Dichlorodiphenyltrichloroethane)
AB07944741-01	67-97-0	Vitamin D3
AB07944749-01	116-31-4	trans-Retinal (Vitamin A aldehyde)
AB07944785-01	111-30-8	Glutaraldehyde (Glutaric dialdehyde)
AB07944787-01	548-62-9	Hexamethyl-p-rosaniline chloride (Gentian violet)
AB07944800-01	3018-12-0	Dichloroacetonitrile
AB07976081-01	2451-62-9	Tris(2,3-epoxypropyl)isocyanurate
AB07980929-01	70-30-4	Hexachlorophene
AB07980958-01	66-71-7	o-Phenanthroline
AB07980986-01	532-27-4	Chloroacetophenone
AB07981015-01	4074-88-8	Diethylene glycol diacrylate
AB07981053-01	81-55-0	1,8-Dihydroxy-4,5-dinitroanthraquinone
AB07981076-01	58-14-0	Pyrimethamine
AB07981167-01	2465-27-2	Auramine
AB07981244-01	99-98-9	N,N-Dimethyl-p-phenylenediamine
AB07981253-01	764-42-1	Fumaronitrile
AB07981260-01	71-58-9	Medroxyprogesterone acetate
AB07981274-01	33229-34-4	HC blue 2
AB07981291-01	50-55-5	Reserpine
AB07981301-01	118-75-2	Chloranil
AB07981317-01	13048-33-4	1,6-Hexanediol diacrylate
AB07981330-01	4252-78-2	2,2',4'-Trichloroacetophenone
AB07981368-01	120-80-9	Catechol (1,2-Benzenediol)
AB07981443-01	434-13-9	Lithocholic acid
AB07981616-01	83-88-5	Riboflavin
AB07981816-01	569-61-9	Basic red 9 (p-Rosaniline HCl) (C.I. 42500)
AB07985231-01	6459-94-5	Acid red 114 (C.I. 23635)
AB07985248-01	97-23-4	2,2'-Methylenebis-(4-chlorophenol)
AB07985289-01	1675-54-3	Bisphenol A diglycidyl ether
AB07985291-01	156-10-5	p-Nitrosodiphenylamine
AB07985305-01	992-59-6	Direct red 2 (C.I. 23500)
AB07985346-01	93-05-0	N,N-Diethyl-p-phenylenediamine
AB07985386-01	133-06-2	Captan
AB07990320-01	53-19-0	o,p'-DDD
AB07990339-01	137-30-4	Ziram
AB07990348-01	55-55-0	N-Methyl-p-aminophenol sulfate

Table 6, continued

Sample ID	CAS	Name
AB07990352-01	140-49-8	4'-(Chloracetyl)acetanilide
AB07990366-01	1239-45-8	Ethidium bromide
AB08001062-01	434-07-1	Oxymetholone
AB08001092-01	3524-68-3	Pentaerythritol Triacrylate
AB08001212-01	12789-03-6	Chlordane, technical grade
AB08001240-01	60348-60-9	Pentabromodiphenyl Ether (PBDE 99) *
AB08001301-01	20562-02-1	a-Solanine
AB08001481-01	70-25-7	N-Methyl-N'-nitro-N-nitrosoguanidine
AB08001574-01	630-16-0	1,1,1,2-Tetrabromoethane
AB08002778-01	148-24-3	8-Hydroxyquinoline
AB08002816-01	2437-29-8	Malachite Green Oxalate
AB08002899-01	7789-12-0	Sodium Dichromate Dihydrate
AB08006114-01	538-75-0	1,3-Dicyclohexylcarbodiimide
AB08006152-01	96-69-5	4,4-Thiobis(6-t-butyl-m-cresol)
AB08006184-01	67-20-9	Nitrofurantoin
AB08006278-01	14371-10-9	Cinnamaldehyde (trans), neat
AB08006839-01	57-97-6	7,12-Dimethylbenz(a)anthracene
AB08007015-01	6317-18-6	Methylene bis(thiocyanate)
AB08007268-01	121-54-0	Benzethonium chloride
AB08007325-01	57-63-6	Ethinyl estradiol
AB08007382-01	5743-04-4	Cadmium acetate, dihydrate
AB08007516-01	518-82-1	Emodin (bulk)
AB08007549-01	23541-50-6	Daunomycin HCL
AB08007613-01	77-47-4	Hexachlorocyclopentadiene
AB08007627-01	793-24-8	n-(1,3-Dimethylbutyl)-n`-phenyl-p-phenylenediamine
AB08080834-01	137-26-8	Tetramethylthiuram disulfide
AB08080839-01	8001-28-3	Croton oil
AB08080841-01	79-94-7	3,3',5,5'-Tetrabromo bisphenol A
AB08080842-01	6088-51-3	6-Hydroxy-2-naphthyl disulfide (DDD)
AB08080844-01	7487-94-7	Mercuric chloride
AB08080867-01	95-84-1	2-Amino-4-methylphenol
AB08080895-01	613-13-8	2-Aminoanthracene
AB08080899-01	91-44-1	7-Diethylamino-4-methylcoumarin
AB08080916-01	6112-76-1	6-Mercaptopurine monohydrate
AB08080928-01	2107-76-8	5,7-Dihydroxy-4-methylcoumarin
AB08082706-01	138-89-6	N,N-Dimethyl-p-nitrosoaniline
AB08082716-01	21829-25-4	Nifedipine
AB08082717-01	1260-17-9	Carminic acid
AB08097184-01	58-54-8	Ethacrynic acid
AB08097232-01	53-86-1	Indomethacin
AB08548260-01	12083-48-6	Bis(cyclopentadienyl)vanadium chloride
AB08548268-01	72-54-8	Rhothane (TDE)
AB08548314-01	17831-71-9	Tetraethylene glycol diacrylate
AB08548327-01	143-50-0	Chlordecone (Kepone)
AB08548342-01	101-90-6	Diglycidyl resorcinol ether

Table 6, continued

Sample ID	CAS	Name
AB08548348-01	101-96-2	N,N'-Di-sec-butyl-p-phenylenediamine
AB08582918-01	11024-24-1	Digitonin
AB08582920-01	101-72-4	N-Isopropyl-N'-phenyl-p-phenylenediamine
AB08582926-01	4196-86-5	2,2-Bis[(benzoyloxy)methyl]dibenzoate propanediol
AB08582927-01	4901-51-3	2,3,4,5-Tetrachlorophenol
AB08582929-01	55-86-7	Nitrogen mustard HCl
AB08582940-01	52417-22-8	9-Aminoacridine HCl H2O
AB08582948-01	63-92-3	Phenoxybenzamine HCl
AB08582961-01	5493-45-8	1,2-Cyclohexanedicarboxylic acid, bis(oxiranylmethyl) ester
AB08582976-01	26530-20-1	2-Octyl-3-isothiazolone
AB08582978-01	129-15-7	2-Methyl-1-nitroanthraquinone
AB08582980-01	97-24-5	2,2'-Thiobis(4-chlorophenol)
AB08582982-01	8001-35-2	Toxaphene
AB08582984-01	86-50-0	Azinphosmethyl (Gusathion)
AB08583007-01	133-18-6	Phenethyl anthranilate
AB13681039-01	39025-23-5	Guggulsterones Z
AB13681051-01	39025-24-6	Guggulsterones E
AB13681076-01	18642-44-9	Actein
NCGC00021272-05	97-18-7	2,2'-Thiobis(4,6-dichlorophenol)
NCGC00021766-03	8003-22-3	D & C yellow no. 11
NCGC00022037-04	71-58-9	Medroxyprogesteroneacetate
NCGC00022185-07	57-83-0	Progesterone
NCGC00022282-03	2062-78-4	Pimozide
NCGC00023188-06	58-14-0	Pyrimethamine
NCGC00023458-07	396-01-0	Triamterene
NCGC00023945-05	30516-87-1	3'-Azido-3'-deoxythymidine
NCGC00024135-11	53-86-1	Indomethacin
NCGC00024253-05	113-52-0	Imipramine_HCl
NCGC00024415-36	25316-40-9	Adriamycin, hydrochloride
NCGC00024910-05	66-81-9	Cycloheximide
NCGC00024928-06	54965-24-1	Tamoxifen citrate
NCGC00025000-05	65277-42-1	Ketoconazole
NCGC00025005-07	446-72-0	Genistein
NCGC00025057-09	520-36-5	Apigenin
NCGC00025060-06	59-05-2	Methotrexate
NCGC00025114-05	1675-54-3	Bisphenol A diglycidyl ether
NCGC00025179-05	84371-65-3	Mifepristone
NCGC00025258-05	N/A	GW7647
NCGC00025331-07	303-45-7	Gossypol
NCGC00076924-01	87-17-2	salicylanilide
NCGC00080412-01	533-74-4	Dazomet
NCGC00089748-06	63-92-3	Phenoxybenzamine hydrochloride
NCGC00090752-08	65646-68-6	N-(4-Hydroxyphenyl)retinamide
NCGC00090757-02	148-82-3	Melphalan
NCGC00090788-04	1948-33-0	t-Butylhydroquinone

Table 6, continued

Sample ID	CAS	Name
NCGC00090792-04	91-53-2	Ethoxyquin
NCGC00090797-03	20830-75-5	Digoxin
NCGC00090832-02	207-08-9	Benzo(k)fluoranthene
NCGC00090836-04	446-86-6	Azathioprine
NCGC00090851-04	320-67-2	5-Azacytidine
NCGC00090866-02	205-99-2	Benzo(b)fluoranthene
NCGC00091032-03	427-51-0	Cyproterone acetate
NCGC00091034-03	133-06-2	Captan
NCGC00091035-02	789-02-6	DDT, o,p'
NCGC00091112-02	548-62-9	Hexamethyl-p-rosaniline chloride
NCGC00091143-04	87-86-5	Pentachlorophenol
NCGC00091195-04	70-30-4	Hexachlorophene
NCGC00091249-02	33229-34-4	HC blue 2
NCGC00091250-04	50-55-5	Reserpine
NCGC00091272-06	434-13-9	Lithocholic acid
NCGC00091325-05	97-23-4	2,2'-Methylene-bis (4-chlorophenol)
NCGC00091332-02	156-10-5	p-Nitrosodiphenylamine
NCGC00091374-06	53-19-0	o,p'-DDD
NCGC00091384-02	140-49-8	4-(Chloroacetyl)acetanilide
NCGC00091432-02	3524-68-3	Pentaerythritol triacrylate
NCGC00091433-02	57-74-9	Chlordane
NCGC00091497-02	538-75-0	Dicyclohexylcarbodiimide
NCGC00091503-02	96-69-5	4,4'-Thiobis(6-tert-butyl-m-cresol)
NCGC00091518-02	57-97-6	7,12-Dimethylbenzanthracene
NCGC00091525-02	6317-18-6	Methylene bis(thiocyanate)
NCGC00091528-02	121-54-0	Benzethonium chloride
NCGC00091540-05	518-82-1	Emodin
NCGC00091563-04	137-26-8	Thiram
NCGC00091595-02	90-43-7	o-Phenylphenol
NCGC00091609-02	91-93-0	3,3'-Dimethoxybenzidine-4,4'-diisocyanate
NCGC00091750-02	72-54-8	Tetrachlorodiphenylethane
NCGC00091775-02	534-52-1	4,6-Dinitro-o-cresol
NCGC00091814-02	101-96-2	N,N'-Di-sec-butyl-p-phenyldiamine
NCGC00091875-02	26530-20-1	2-Octyl-3-isothiazolone
NCGC00091883-04	86-50-0	Azinphosmethyl
NCGC00091887-04	309-00-2	Aldrin;Aldrin
NCGC00092353-03	82640-04-8	Raloxifene hydrochloride
NCGC00093553-06	54-62-6	Aminofolic acid, 4-
NCGC00093877-04	138090-06-9	(R,R)-cis-Diethyltetrahydro-2,8-chrysenediol
NCGC00094190-04	50-65-7	Niclosamide
NCGC00094248-05	630-60-4	Ouabain
NCGC00094382-04	83-79-4	Rotenone
NCGC00094423-07	97-77-8	Tetraethylthiuram disulfide
NCGC00094539-03	1918-16-7	Propachlor
NCGC00094545-03	2312-35-8	Propargite
NCGC00094560-03	1897-45-6	Chlorothalonil

Table 6, continued

Sample ID	CAS	Name
NCGC00094842-05	480-40-0	chrysin
NCGC00095272-03	149-29-1	Patulin
NCGC00142516-03	1397-94-0	Antimycin A
NCGC00142623-02	71-63-6	Digitoxin
NCGC00159417-03	3380-34-5	Triclosan
NCGC00159424-03	97-74-5	Thiodicarbonic diamide ([(H2N)C(S)]2S), tetramethyl-
NCGC00159555-02	293754-55-9	T0901317
NCGC00161622-02	50-76-0	Actinomycin D
NCGC00162403-02	303-47-9	Ochratoxin A
NCGC00163107-03	338404-52-7	CITCO
NCGC00163705-02	14816-18-3	Phoxim
NCGC00163706-01	124495-18-7	Quinoxyfen
NCGC00163711-01	300-76-5	Naled
NCGC00163712-01	20018-09-1	Diiodomethyl p-tolyl sulfone
NCGC00163713-01	N/A	Conazole Mix IV (584:80,464:20)
NCGC00163725-01	79622-59-6	Fluazinam
NCGC00163729-04	12427-38-2	Mancozeb
NCGC00163732-01	113507-06-5	Moxidectin
NCGC00163740-01	122453-73-0	Chlorfenapyr
NCGC00163743-01	63333-35-7	Bromethalin
NCGC00163750-01	66841-25-6	Tralomethrin
NCGC00163780-01	N/A	Milbemectin (A mixture of >=70% Milbemcin A4, & <=30% Milbemycin A3)
NCGC00163783-01	67485-29-4	Hydramethylnon
NCGC00163790-01	123997-26-2	Eprinomectin
NCGC00163795-01	121552-61-2	Cyprodinil
NCGC00163818-01	131860-33-8	Azoxystrobin
NCGC00163821-01	9003-13-8	Butoxypolypropylene glycol
NCGC00163823-01	72490-01-8	Fenoxycarb
NCGC00163828-02	69327-76-0	Buprofezin
NCGC00163864-01	62924-70-3	Flumetralin
NCGC00163879-01	2642-71-9	Azinphos ethyl;Azinphosethyl
NCGC00163880-01	126-72-7	Tris(2,3-dibromopropyl)phosphate
NCGC00163895-01	175013-18-0	Pyraclostrobin
NCGC00163896-01	143390-89-0	Kresoxim-methyl
NCGC00163897-01	2032-65-7	Methiocarb
NCGC00163909-01	76-87-9	Fentin hydroxide
NCGC00163915-01	134098-61-6	Fenpyroximate
NCGC00163939-01	28772-56-7	Bromadiolone
NCGC00163942-01	56-35-9	Tributyltin oxide
NCGC00163962-01	2939-80-2	Captafol
NCGC00163965-01	14484-64-1	Ferbam
NCGC00163974-01	191-24-2	Benzo[g,h,i]perylene
NCGC00163987-01	117-80-6	2,3-Dichloro-1,4-naphthoquinone
NCGC00164034-01	101-20-2	Triclocarban
NCGC00164057-01	52-51-7	2-Bromo-2-nitro-1,3-propanediol

Table 6, continued

Sample ID	CAS	Name
NCGC00164061-01	97-00-7	Dinitrochlorobenzene
NCGC00164088-01	3567-69-9	C.I. Acid red 14
NCGC00164095-01	111-82-0	Dodecanoic acid, methyl ester
NCGC00164096-01	688-73-3	TRIBUTYLTIN
NCGC00164104-01	15625-89-5	Trimethylolpropane triacrylate
NCGC00164110-01	4638-48-6	5-chlorosalicylanilide
NCGC00164120-01	31519-22-9	1,4-dihydroxy-2-naphthoic acid
NCGC00164125-01	2767-54-6	Triethyltin
NCGC00164126-01	1034-01-1	Octyl gallate
NCGC00164162-01	124-64-1	Tetrakis(hydroxymethyl)phosphonium chloride
NCGC00164172-01	119-47-1	Phenol, 2,2'-methylenebis[6-(1,1-dimethylethyl)-4-methyl-
NCGC00164179-01	4342-36-3	Tributyltin benzoate
NCGC00164182-01	78-04-6	1,3,2-Dioxastannepin-4,7-dione, 2,2-dibutyl-
NCGC00164186-01	1461-22-9	Stannane, tributylchloro-
NCGC00164192-01	2650-18-2	Acid Blue 9
NCGC00164203-01	10222-01-2	DBNPA
NCGC00164206-01	2634-33-5	Benzisothiazolin-3-one
NCGC00164211-01	5930-28-9	4-amino-2,6-dichlorophenol
NCGC00164222-01	87-56-9	2-Butenoic acid, 2,3-dichloro-4-oxo-, (2Z)-
NCGC00164224-01	88-30-2	3-trifluoromethyl-4-nitrophenol
NCGC00164228-01	122-37-2	phenol,4-(phenylamino)-
NCGC00164258-01	59865-13-3	Cyclosporin A
NCGC00164263-01	161326-34-7	Fenamidone
NCGC00164283-01	57-09-0	Cetyltrimethylammonium bromide
NCGC00164294-01	13121-70-5	Cyhexatin (Plictran)
NCGC00164304-01	85509-19-9	NuStar
NCGC00164309-01	96489-71-3	Pyridaben
NCGC00164348-01	683-18-1	Stannane, dibutyldichloro-
NCGC00164354-01	639-58-7	Triphenyltin chloride
NCGC00164363-01	1067-33-0	Dibutyltin diacetate
NCGC00164373-01	88-85-7	Phenol, 2-(1-methylpropyl)-4,6-dinitro-
NCGC00164376-01	55406-53-6	Carbamic acid, butyl-, 3-iodo-2-propynyl ester
NCGC00164385-01	50-41-9	Clomiphene citrate
NCGC00164397-01	52-01-7	Spironolactone
NCGC00164399-01	65213-48-1	4-Hydroxytamoxifen
NCGC00164410-01	26027-38-3	Igepal CO-210
NCGC00164411-01	17095-24-8	2,7-Naphthalenedisulfonic acid, 4-amino-5-hydroxy-3,6-bis[[4-[[2-(sulfooxy)ethyl]sulfonyl]phenyl]azo]-, tetrasodium salt
NCGC00164412-01	28961-43-5	Poly(oxy-1,2-ethanediyl), .alpha.-hydro-.omega.-[(1-oxo-2-propenyl)oxy]-, ether with 2-ethyl-2-(hydroxymethyl)-1,3-propanediol (3:1)
NCGC00164424-01	62-38-4	Phenylmercuric acetate
NCGC00164425-01	54-64-8	((o-Carboxyphenyl)thio)ethylmercury sodium salt
NCGC00164436-01	N/A	Emamectin benzoate
NCGC00164444-01	13014-03-4	Copper, bis(8-quinolinolato-N1,O8)-,

Table 6, continued

Sample ID	CAS	Name
NCGC00164445-01	97322-87-7	Troglitazone
NCGC00164446-01	74772-77-3	Ciglitizone
NCGC00164454-01	57960-19-7	Acequinocyl
NCGC00164456-01	137-30-4	Ziram
NCGC00164463-01	83657-18-5	Diniconazole-M
NCGC00168290-01	71751-41-2	Abamectin
NCGC00168297-01	120116-88-3	Cyazofamid
NCGC00168306-01	131807-57-3	Famoxadone
NCGC00168313-01	133-07-3	Folpet
NCGC00168337-01	21564-17-0	TCMTB
NCGC00168338-01	119168-77-3	Tebufenpyrad

References

Hill AV. 1910. The possible effects of the aggregation of the molecules of haemoglobin on its dissociation curves. J Physiol (London) 40: 4-7.

Huang R, Southall N, Xia M, Cho MH, Jadhav A, Nguyen DT, et al. 2009. Weighted feature significance: a simple, interpretable model of compound toxicity based on the statistical enrichment of structural features. Toxicol Sci 112(2): 385-393.

Inglese J, Auld DS, Jadhav A, Johnson RL, Simeonov A, Yasgar A, et al. 2006. Quantitative high-throughput screening: a titration-based approach that efficiently identifies biological activities in large chemical libraries. Proc Natl Acad Sci U S A 103(31): 11473-11478.

Judson R, Richard A, Dix DJ, Houck K, Martin M, Kavlock R, et al. 2009. The toxicity data landscape for environmental chemicals. Environ Health Perspect 117(5): 685-695.

PubChem. 2007. NTP compound collection. Available: http://www.ncbi.nlm.nih.gov/sites/entrez?db=pcsubstance&term=niehs [accessed 15 February 2008].

PubChem. 2009. Tox21 Phase I compound collection. Available: http://www.ncbi.nlm.nih.gov/sites/entrez?db=pcsubstance&term=Tox21_EPA1_v1a [accessed 12 September 2009].

Xia M, Huang R, Sun Y, Semenza GL, Aldred SF, Witt KL, et al. 2009. Identification of chemical compounds that induce HIF-1alpha activity. Toxicol Sci 112(1): 153-163.

Xia M, Huang R, Witt KL, Southall N, Fostel J, Cho MH, et al. 2008. Compound cytotoxicity profiling using quantitative high-throughput screening. Environ Health Perspect 116(3): 284-291.
